# A Systematic Review on the Safety and Efficacy of COVID-19 Vaccines Approved in Saudi Arabia

**DOI:** 10.3390/vaccines11020281

**Published:** 2023-01-28

**Authors:** Thekra Ali Alhandod, Syed Imam Rabbani, Mansour Almuqbil, Sultan Alshehri, Syed Arif Hussain, Nasser Fawzan Alomar, Manzoor Ahmad Mir, Syed Mohammed Basheeruddin Asdaq

**Affiliations:** 1 Department of Pharmacology and Toxicology, College of Pharmacy, Qassim University, Buraydah 51452, Saudi Arabia; 2 Department of Clinical Pharmacy, College of Pharmacy, King Saud University, Riyadh 11451, Saudi Arabia; 3 Department of Pharmaceutical Sciences, College of Pharmacy, AlMaarefa University, Riyadh 13713, Saudi Arabia; 4 Respiratory Care Department, College of Applied Sciences, Almaarefa University, Riyadh 13713, Saudi Arabia; 5 Equame Scientific and Research Center, Riyadh 13713, Saudi Arabia; 6 Department of Bioresources, School of Biological Sciences, University of Kashmir, Srinagar 190006, India; 7 Department of Pharmacy Practice, College of Pharmacy, AlMaarefa University, Riyadh 13713, Saudi Arabia

**Keywords:** COVID-19 vaccines, Saudi Arabia, systemic review, safety, efficacy

## Abstract

Comprehensive safety and efficacy studies of COVID-19 vaccines might reduce the apprehension of the general population about the adverse reactions and duration of protection offered by them. The study aimed to conduct a systemic review on the four COVID-19 vaccines (AstraZeneca, Pfizer, Moderna, and Janssen) approved in Saudi Arabia. The study was conducted by reviewing the published articles from electronic databases such as PubMed, Embase, Cochrane Library and Web of Science using the search terms “COVID-19”, “Vaccine”, “Safety”, “Efficacy” and “Human trials” and as per the standard guidelines for systemic review. The review analyzed eighteen articles and the data from them were evaluated to analyze the safety and efficacy of the vaccines in different groups of population such as males, females, those above 18 years and people with co-morbidities. The common local reactions observed after vaccination were pain at the site of injection (40–70%), redness (16–30%), swelling (18–39%) and tenderness (20–40%). The systemic reactions reported were fever (40–60%), chills (12–23%), fatigue (44–65%), headache (30–42%) and muscle pain (15–40%). The efficacy was observed to be above the threshold value (60%) stipulated by the WHO. However, precautions need to be followed while vaccinating special groups of population such as those that are pregnant, lactating or experiencing severe illness. Additionally, the rare and serious adverse events reported remotely after vaccination need more studies.

## 1. Introduction

The available information has indicated that severe acute respiratory syndrome coronavirus 2 (SARS-CoV-2) was first reported in the Wuhan province of China. The infection very rapidly spread to other parts of the world attaining a state of a pandemic. Present status indicates that this disease has affected millions of people worldwide and the situation remains a pandemic globally [1]. The disease has shown significant mortality, especially among the most vulnerable population such as the elderly and patients with co-morbidities. Currently, the second wave of COVID-19 infection has produced havoc and has already caused millions of deaths [2].

Many medical interventions are being tried to treat COVID-19. Some of them have shown promising results while others failed to achieve significant effects [3]. Immunizing a significant proportion of the population is considered one of the better approaches to achieving herd immunity [4].

Studies have suggested that vaccines play a vital role in controlling diseases caused by pathogens, especially those attaining the capacity of human-to-human transmission [5]. Isolation, quarantine, and containment might limit the outbreak but vaccinating the population is reported to convert the pandemic condition into a manageable endemic disease [6]. Further, vaccination reduces morbidity and mortality in the public, thereby minimizing the damaging effects on society, including economic burdens [7]. Therefore, efforts are needed to promote the widespread acceptance of vaccination programs among the public to limit the consequences of any future pandemics.

Accumulating evidence from the literature on vaccines suggests that these prophylactic agents induce mostly minor and manageable side effects in the general population. Although, some rare and potentially fatal reactions such as hepatitis, renal failure, and thromboembolic events were reported, their incidences have not crossed the limits observed with any other medical interventions [8]. Besides, these complications were related to their design, manufacturing technique, genetic predisposition, or comorbidities in the patients, which can be controlled with effective monitoring mechanisms involving patients, medical supervisors, researchers, and manufacturers [9].

Several pharmaceutical companies in collaboration with the research centers have manufactured vaccines and are currently testing them in different phases of trials. Although the studies in most of the cases have not yet reached a logical conclusion, considering the pandemic situation, the World Health Organization has given emergency authorization EUA for some of the vaccines to be used by the public [10].

The vaccines developed by Pfizer, Moderna, AstraZeneca, Sputnik, and Sinopharm have started to market their products in countries that gave their approval [4,10]. In most cases, the vaccines are known to produce mild symptoms such as fever, sore throat, muscle ache, and chills [11]. However, in rare instances, the vaccine has caused a severe form of allergic reaction such as anaphylaxis [12]. Recently, it has been reported that after COVID-19 vaccination, a serious type of pathological blood clotting occurred in some people, leading to hospitalization and mortality. Research suggests that the occurrence of these reactions depends on several factors such as genetic variations, age, and the disease states of the patients [13,14].

Considering these reports, the WHO has given a statutory warning for using the vaccine cautiously in people who are known to suffer from any allergic issues, and a few countries have also put a temporary ban on the use of certain COVID-19 vaccines [15]. In addition, the safety of these vaccines in pregnancy as well as in children under 18 years of age and severely ill patients is not yet established. As the vaccination program is expanded to different sections of society, newer and previously unreported adverse reactions/events are being recorded. A comprehensive analysis of the data from several studies could be an essential tool to establish the safety and efficacy of the COVID-19 vaccine [16]. Hence, this study evaluated the scientific data of four approved vaccines of the Kingdom of Saudi Arabia, AstraZeneca, Pfizer, Moderna, and Janssen, and reviewed their safety and efficacy profiles.

## 2. Materials and Methods

The Preferred Reporting Items for Systematic Reviews and Meta-Analyses (PRISMA) guidelines were followed during the preparation of this systematic review [17]. The steps mentioned by Khan et al. for conducting the systemic review were followed in this study [18].

### 2.1. Literature Search Strategy

An electronic literature search of PubMed, SCOPUS, Web of Science, and BIOSIS was carried out from January 2021 to September 2022 by using the following keywords: COVID-19 vaccine OR Coronavirus vaccine OR Corona vaccine AND Safety AND Efficacy AND Clinical Trials OR Human Trials AND Approved.

### 2.2. Study Selection

Authors involved in this study independently screened the literature search results for four COVID-19 vaccines viz., AstraZeneca (ChAdoxin CoV-19), BioNTech/Pfizer Vaccine (mRNA-BNT162b2), Moderna Vaccine (mRNA-1273) and Janssen Vaccine (JNJ-78436735Ad26) approved in Saudi Arabia [19]. The eligibility screening was performed in two steps: the first step was to screen titles and abstracts of the retrieved records and the second step was to screen full-text articles of abstracts selected in the first step. Discussions were made with the supervisor and/or subject expert to resolve any discrepancies that arise from the findings.

### 2.3. Eligibility Criteria

Studies satisfying the following criteria were included in this review:

- Articles are written in the English language containing detailed information about the type of COVID-19 vaccines and the number of volunteers with their consent.
- Cross-sectional studies that were published in the last three years (2019–2022).
- Studies in which human volunteers were used for COVID-19 vaccine testing.
- Studies evaluating the safety and efficacy of the COVID-19 vaccine, including the dose, duration, presence of adverse reactions, and a clear procedure to determine the effectiveness.
- Published articles in reputed journals giving comprehensive information about statistics and their significance.

Studies that did not fit the above-mentioned eligibility criteria were excluded. Additionally, the studies with overlapped data sets, duplicated reports, and studies with data that could not be extracted were excluded [20].

### 2.4. Data Extraction

Authors through a pre-organized data extraction sheet retrieved the data independently. The extracted data included characteristics of the study design, characteristics of the study population, and data on the study outcomes.

### 2.5. Quality Assessment

The Newcastle–Ottawa scale tool for measuring the risk of bias assessment of cross-sectional studies was used. This tool comprises various domains such as sampling plan, statistical analysis description, and outcomes [21]. Authors blindly evaluated the quality of included studies and any disagreements were settled through discussion with the supervisor/subject expert.

## 3. Results

### 3.1. Extraction of the Scientific Data for the Approved COVID-19 Vaccines

**Figure 1:**
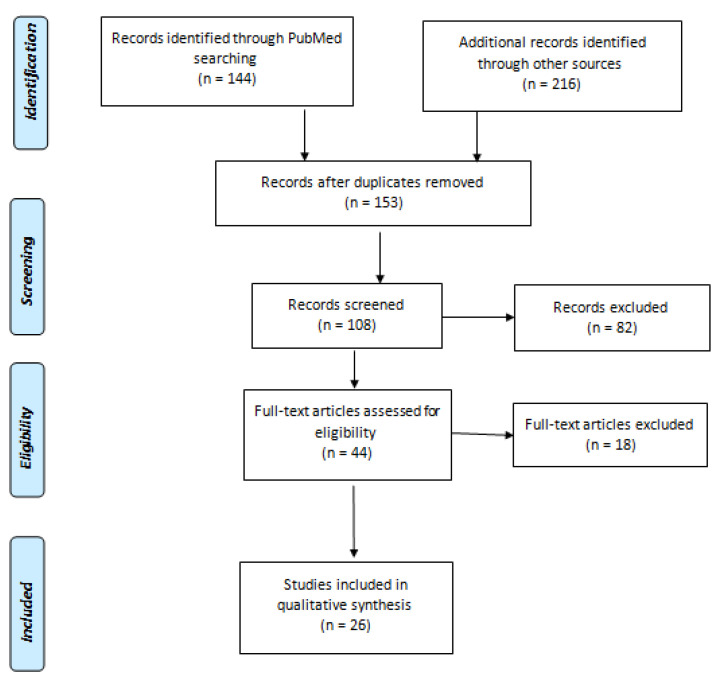
PRISM flow diagram used to select the reported studies for review.

A total 360 articles were reviewed from different scientific search engines. After analyzing duplicates of the data, 153 articles were selected. Some of the articles were removed due to inadequate data, while some were excluded since full-length article information was missing. A detailed summary of the extraction of scientific data from the literature is represented in Figure 1.

All included articles were considered of good quality. This assessment was done based on the Newcastle–Ottawa scale tool. A total of 11 articles scored ‘9 stars’, while 15 articles got ‘7 stars’. All articles were granted stars depending on the information present in them, such as the representation of the sample, sample size, response rate, assessment of exposure, study controls, and assessment of outcomes, including the statistical tests.

### 3.2. Characteristics of Participants Enrolled for COVID-19 Vaccines Testing [22,23,24,25]

The available data suggested that the four vaccines approved in Saudi Arabia have been tested on both males and females and on different age-grouped participants. The AstraZeneca vaccine was tested on 60.29% males and 39.7% females, while Pfizer’s was tested on 51.07% males and 48.9% females. Similarly, the Moderna vaccine was tested on 52.22% males and 47.74% females, and Janssen’s on 55.14% males and 44.85% females.

**Figure 2:**
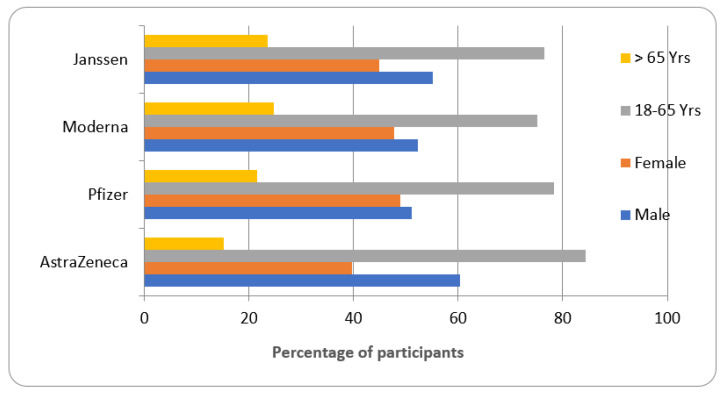
Different groups of participants tested for COVID-19 vaccines.

In the age groups, different proportions of participants are included for AstraZeneca (84.4% were 18–65 years, 15.25% were above 65 years), Pfizer (78.41% were 18–65 years and 21.58% were above 65 years), Moderna (75.17% were 18–65 years and 24.82% were above 65 years) and Janssen (76.53% were 18–65 years and 23.49% were above 65 years) (Figure 2).

### 3.3. Common Local Reactions after COVID-19 Vaccination [26,27,28,29,30,31,32,33]

**Figure 3:**
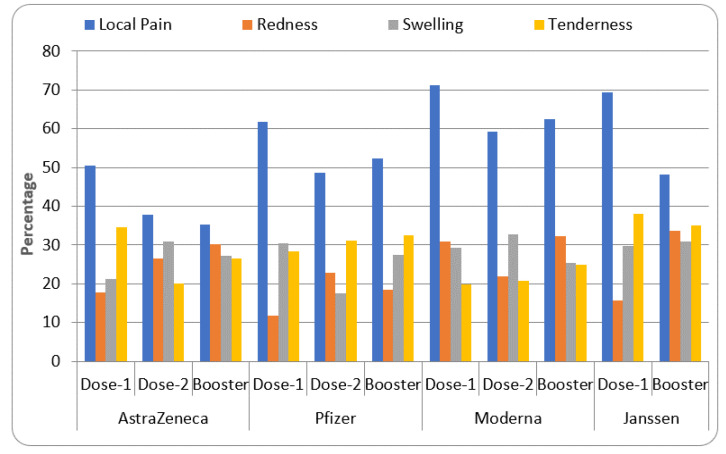
Common local reactions after COVID-19 vaccination.

Figure 3 represents the common local reactions observed after COVID-19 vaccination. Most of the population after their first dose indicated ‘local pain’ at the site of injection as the most frequent local reaction (AstraZeneca—50.6%, Pfizer—61.8%, Moderna—71.2%, and Janssen—69.5%). The second-most common local reaction after the first dose was tenderness (AstraZeneca—34.5%, Janssen—38.1%), followed by swelling—30.5% (Pfizer), and redness—30.8% (Moderna).

These reactions were found to be increased after the second dose of the vaccine, except for local pain (AstraZeneca—37.8%, Pfizer—48.6%, Moderna—59.2%), tenderness—20% (AstraZeneca), swelling—17.5% (Pfizer) and redness—21.9% (Moderna). The local reaction after booster dose administration indicated more incidences of redness (AstraZeneca, Moderna, and Janssen), swelling (Janssen) and tenderness (Pfizer and Moderna) compared to previous dosing. However, lower frequencies of local pain (AstraZeneca and Janssen), swelling (Moderna), and tenderness (Janssen) were observed with the booster dose compared to the preceding dosage.

### 3.4. Common System Reactions after COVID-19 Vaccination [27,29,32,34,35,36]

The five common systemic reactions after COVID-19 vaccination observed were fever, chills, headache, muscle pain, and fatigue. The AstraZeneca vaccine was found to produce fatigue (50.7%) in most people, followed by headache (41.9%) and fever (38.9%). These reactions were found to be reduced after the second dose except for fever (42.8%). Pfizer vaccine produced fatigue (61.8%) and fever (56.9%) in many recipients after the first dose and these were slightly increased after the second dose (fatigue—65.8% and fever—57.2%).

The Moderna vaccine data showed the highest tendency of causing fever (40.9%) and fatigue (38.5%) in the population after the first dose. These reactions were found to be increased after the second dose, including fatigue (49.2%) and fever (52.5%). Single-dose jabs of the Janssen vaccine caused fatigue (43.8%), muscle pain (39.7%), and fever (33.9%) as the major systemic side effects in people.

**Figure 4:**
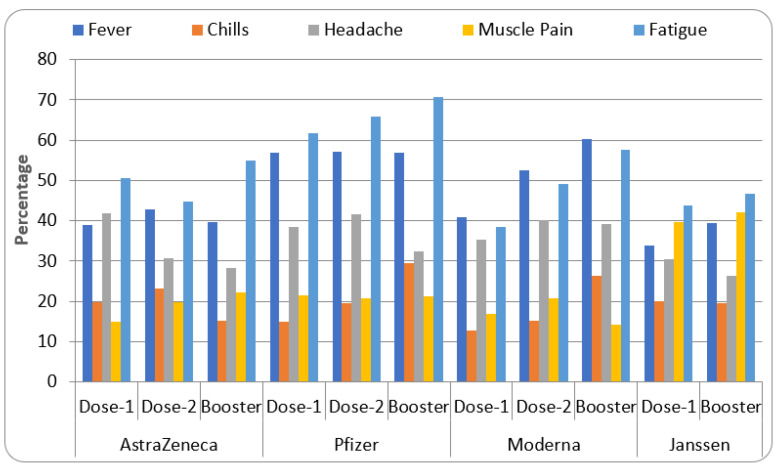
Common system reactions after COVID-19 vaccination.

In addition, the second dose of the COVID-19 vaccine caused more reactions such as ‘chills’ (AstraZeneca—23.1%, Pfizer—19.5%, and Moderna—15.2%) when compared to the first dose. Similarly, muscle pain (AstraZeneca—19.7%, Moderna—20.8%), and headache (Pfizer—41.6%, Moderna—40.1%) were found to be more severe than the first dose. The effect of booster dose on systemic reactions suggested an increase in fever (Moderna and Janssen), chills (Pfizer and Moderna), muscle pain (AstraZeneca and Janssen), and fatigue (all approved vaccines) compared to previous doses. On the other hand, occurrences of chills (AstraZeneca and Janssen), headache (AstraZeneca and Janssen), and muscle pain (Pfizer and Moderna) were found to be reduced with booster dose administration compared to previous dosages (Figure 4).

### 3.5. Efficacy of COVID-19 Vaccine in Different Groups of Participants [26,28,30,33,37,38,39]

**Figure 5:**
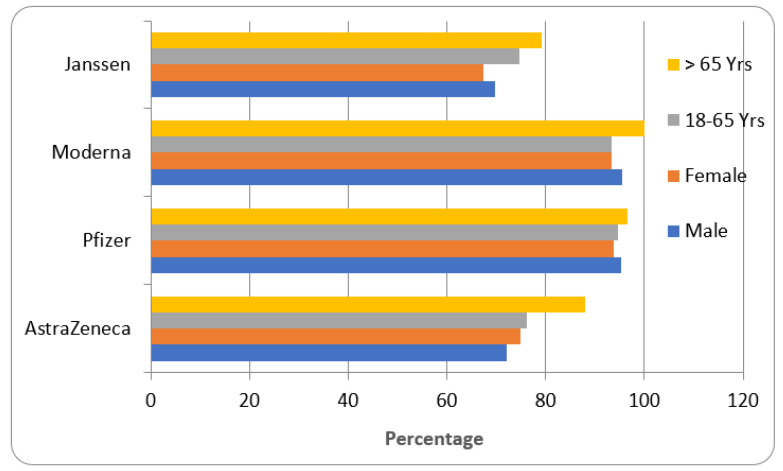
Efficacy of COVID-19 vaccine.

The efficacy of different COVID-19 vaccines is represented in Figure 5. The efficacy of the vaccine in males was found to be 72.1% for AstraZeneca, 95.3% for Pfizer, 95.5% for Moderna, and 69.8% for Janssen, while in females the efficacy was 74.8% for AstraZeneca, 93.9% for Pfizer, 93.4% for Moderna and 67.3% for Janssen.

Among the 18–65 year-old population, the highest efficacy was observed with Pfizer (94.6%), followed by Moderna (93.4%), AstraZeneca (76.2%), and Janssen (74.7%). All four vaccines have shown maximum efficacy in those above 65 years of age (AstraZeneca—88.1%, Pfizer—96.5%, Moderna—100%, and 79.1% Janssen).

## 4. Discussion

The data from the systemic review are represented in Table 1 and Figure 1, Figure 2, Figure 3, Figure 4 and Figure 5. Four COVID-19 vaccines approved in Saudi Arabia were studied to evaluate their safety and efficacy. The study was conducted as per the guidelines of systemic review [19,21]. Characteristics of participants, common local and systemic reactions, efficacy in different groups of the population, and descriptive information about the vaccines are summarized.

According to WHO, vaccination is one of the most effective methods to reduce the chances of COVID-19 infection in the population. The development of vaccines undergoes several stages where the safety and efficacy are established by testing them on different groups of the population. All the vaccines approved for clinical use are required to follow strict safety and efficacy guidelines [11,12]. It has been reported that vaccine safety differs according to the group of volunteers enrolled in the study. The genetic predisposition and type of technology used to develop the vaccine also play an important role in determining efficacy and safety [22].

The available data suggested that the vaccines were tested on different races of the population residing in different regions of the world. The population comprised both genders were tested males and females), while volunteers of age between 18–65 years constituted ≈75% and above 65≈25% (Figure 2). The volunteers categorized as ‘high-risk’ were sufferers of CVS, respiratory, kidney, liver, and neurological diseases and were evaluated for safety and efficacy (Table 1).

According to previous reports, COVID-19 vaccines are known to cause common local reactions (pain at the site of injection, inflammation, warmth, and redness) and systemic reactions (fever, chills, headache, myalgia, and fatigue) [23]. Hence, the present study summarized the percentage of these common local and systemic reactions. The data from the study suggested that the most frequent local reaction for the four approved vaccines in Saudi Arabia is local pain followed by swelling and tenderness (Figure 3), and the most common systemic reaction observed in this study was fatigue followed by high temperature and headache. Most of these reactions increased after the second dose of the vaccines (Figure 4). Administration of the first booster dose of COVID-19 vaccine increased some of the local and systemic reactions but the incidences were found to be slightly higher than the previous dosages (Figure 3 and Figure 4).

The literature review suggested that the administration of vaccines sends out a ‘danger’ signal within minutes or even seconds by the antigen-detecting cells of the body. This rapid reaction, also known as innate immune response, is reported to involve a slew of immune cells that arrive and produce proteins such as cytokines, chemokines, and prostaglandins [23,24].

Cytokines are known to dilate the blood vessels to increase the blood flow resulting in swelling and redness. These proteins can also irritate the nerves causing pain. Additionally, cytokines and chemokines can induce inflammation and pain. The prostaglandins can directly interact with the receptors to cause pain sensation. These reactions in a few individuals do not stop at this stage but continue to cause fever, body aches, joint pain, rashes, and/or headaches [25]. The reactions tend to be more severe after the second or subsequent dose due to the presence of already-synthesized immune cells. Reports also suggested that the development or non-development of these reactions has no relationship with the antibodies’ production [26].

The efficacy data indicated that the AstraZeneca vaccine was found to be effective (>70%) in all the groups of tested participants (Figure 5). This vaccine is vector-based and requires two doses of administration (Table 1). The innovative technology (RNA-based) used in vaccines such as Pfizer and Moderna showed an efficacy of more than 90% in preventing the chance of COVID-19 in the test volunteers (Figure 5). These vaccines are also required to be given in two doses (Table 1). The single jab COVID-19 vaccine (Janssen) is vector-based and showed an efficacy of more than 68% in the tested groups (Figure 5). Among the four vaccines, only Pfizer requires a special storage temperature of −20 to −700 °C (Table 1).

Earlier research revealed that there are four main types of COVID-19 vaccines, such as those containing a whole virus, protein subunit, viral vector, and nucleic acid (DNA/RNA). In whole-virus vaccines, the causative organism is either weakened or its genetic material is destroyed. The administration of such a form of the virus does not support the virus replication but triggers the immune system to develop antibodies [27].

The protein subunit vaccines contain small fragments (such as spike proteins) of the microorganism that are recognized by the antigen-detecting cells to develop the antibodies. The nucleic acid vaccines contain either DNA or RNA that carry specific information for viral proteins (Spike proteins) [28]. These genetic materials, after combining with the host nucleus, direct the production of viral proteins which will be then identified by the immune system to develop antibodies. The vector vaccines contain the harmless virus, called a vector, that carries the genetic information (DNA) for producing viral proteins after combining with the host cells [29].

Amid the four vaccines used for the study, Moderna and Pfizer COVID-19 vaccines were developed from nucleic acid technology and contain mRNA as the genetic component. They require special storage conditions since RNA is more temperature-labile than DNA [27,29]. AstraZeneca and Janssen vaccines are vector-based and carry the genetic information embedded in an adenovirus (Table 1). The mRNA vaccine is an innovation while vector-based vaccines are used traditionally to stimulate the immunological response [22,39]. All four COVID-19 vaccines were found to cross the threshold value of protection (60%) suggested by the WHO. Another interesting observation of the study is that the efficacy of all the vaccines was found to be maximum in people above 65 years of age (Figure 5). One of the reasons suggested for this is that the elderly population of society is more cautious and takes more precautionary measures during outbreaks of any pandemic compared to other age groups [39]. The efficacy was determined by comparing the COVID-19-positive cases detected in recipients administered with either placebo or vaccine [40].

The data from this study summarized some common local and systemic reactions observed after the administration of COVID-19 vaccines (Figure 2 and Figure 3). In addition to the descriptive information given in Table 1, the expansion of vaccination programs in different groups of the population across the globe has shown some rare but serious adverse events. The AstraZeneca vaccine has caused a severe allergic reaction, the sudden swelling of legs, and weight gain [41]. Pfizer vaccine-treated individuals recently reported enlarged lymph nodes, one-sided facial drooping, and life-threatening allergic reactions [42]. Moderna vaccine administration has indicated anaphylaxis, breathing difficulties, and fainting [43]. Janssen’s COVID-19 vaccination reported embolic and thrombotic events in addition to tinnitus [44]. The events/reactions, although occurring remotely, need to be closely monitored and studied to understand the underlying pathology before establishing complete safety. Currently, in many countries, including Saudi Arabia, the second booster dose of the COVID-19 vaccine is not yet approved for the general population. This dose is recommended for patients suffering from a compromised immune system, renal failure, cancer, and patients who have undergone organ transplant [45]. Therefore, it is also essential to analyze the booster dose effects of COVID-19 vaccines in all groups of the population including patients diagnosed with chronic diseases.

## 5. Conclusions

The systemic review of the four COVID-19 vaccines approved in Saudi Arabia indicated some common local and systemic reactions that are frequently seen after any vaccination. Analysis of the data suggested that the approved vaccines provided the required level of efficacy against COVID-19 infection (above 60%), as per the WHO norms. However, as the vaccination program spreads to different sections of society, risky adverse events such as anaphylaxis and pathological blood clotting have been linked to the COVID-19 vaccines. A more detailed and comprehensive study involving different groups of the population including a challenge treatment is essential to establish the complete safety and efficacy of the vaccines. This further becomes vital as some of the adverse effects were found to be more pronounced with the administration of booster doses.

## 6. Future Implication and Limitation of the Study

COVID-19 has affected almost all the countries of the world. The pandemic has caused significant unrepairable damage to the economy and loss of precious lives. In the absence of specific therapeutic interventions, vaccination is the better approach to limiting the spread of disease. However, extensive testing, data analysis, and systemic and meta-analysis of such results are necessary to establish the safety and efficacy of the vaccines.

In one such attempt, the present study conducted a systemic review of the safety and efficacy of four approved COVID-19 vaccines in Saudi Arabia. The study analyzed the published data for these four vaccines. Since the information on vaccines is regularly updated due to the expansion of vaccination programs, the compilation and analysis of such data is essential and could act as the major tool to determine the precise characteristics of vaccines.

## Figures and Tables

**Table 1:** Descriptive information on the four approved COVID-19 vaccines.

Parameters	AstraZeneca [12,25,34]	Pfizer [15,22,30,32]	Moderna [11,21,32,35]	Janssen [8,36,39]
Vaccine type	Vector-based	RNA-based	RNA-based	Vector-based
Manufacturing country	UK	US	US	The Netherlands/US
Dose	0.5 mL	0.3 mL	0.5 mL	0.5 mL
Number of dose and suggested duration between them *	2 (28 days)	2 (21 days)	2 (28 days)	Single dose
Route of administration	Intra-muscular (Preferably deltoid muscle)	Intra-muscular (Preferably deltoid muscle)	Intra-muscular (Preferably deltoid muscle)	Intra-muscular (Preferably deltoid muscle)
Mechanism of action	Adenovirus is used as a vector to carry the genetic code for making viral spike protein by host cells, triggering antibody production.	m-RNA induces the host cell to produce spike proteins, triggering the production of immune cells.	m-RNA induces the host cell to produce spike proteins, triggering the production of immune cells.	Adenovirus is used as a vector to carry the genetic code for making viral spike protein by host cells, triggering antibody production.
Protection post-vaccination	Significant antibody levels can be seen after 14 days	Significant antibody level in blood after 14 days	Significant antibody level in blood after 14 days	Significant antibody level in blood after 14 days
High risk participants	Patients suffering from cardiovascular, respiratory, metabolic, hepatic, renal, haematological and immunocompromised conditions			
Storage temperature	2 to 8 °C	−20 to −70 °C	2 to 8 °C	2 to 8 °C
Specific adverse events	Thrombosis, Anaphylaxis	Lymphadenopathy, Myocarditis	Cholecystitis, Myocardial infarction	Hypersensitivity, Appendicitis
Precautions	Pregnancy, lactation, less than 18 years, severe illness and allergic to vaccine components			

* Recent reports suggest that increasing the gap between two doses enhances efficacy.

## Data Availability

Publicly available datasets were analyzed in this study. This data can be found at https://pubmed.ncbi.nlm.nih.gov/ (accessed on 15 December 2022) and https://scholar.google.com/scholar?hl=en&as_sdt=0%2C5&as_vis=1&q=covid-19+vaccines%2C+safety+and+efficacy&btnG= (accessed on 20 December 2022).
